# Supplementary material for: NADPH Oxidases: The Vital Performers and Center Hubs during Plant Growth and Signaling
Source: Cells. 2020 Feb 13;9(2):437. doi: 10.3390/cells9020437 (PMC7072856; doi:10.3390/cells9020437)
Supplement: Supplementary file 1 [file cells-09-00437-s001.pdf]

Review **Supplementary Material**

Title: NADPH oxidases: the Vital Performers and Center Hubs during Plant Growth and Signaling

Chun-Hong Hu [1,2,†], Peng-Qi Wang [3,†], Peng-Peng Zhang [1], Xiu-Min Nie [1], Bin-Bin Li [1], Li Tai [1], Wen-Ting Liu [1], Wen-Qiang Li [1] and Kun-Ming Chen [1,*]

[1] State Key Laboratory of Crop Stress Biology in Arid Areas, College of Life Sciences, Northwest A&F University, Yangling 712100, Shaanxi, China; ourcarrot@163.com (C.-H.H.); 15256849763@163.com (P.-P.Z.); niexiumin1019@163.com (X.-M.N.); libinbin@nwafu.edu.cn (B.-B.L.); 15591861258@163.com (L.T.); lwt001975@163.com (W.-T.L.); wqli@nwsuaf.edu.cn (W.-Q.L.)

[2] College of Life Science and Agronomy, Zhoukou Normal University, Zhoukou 466000, Henan, China

[3] School of Life Sciences, Tsinghua University, Beijing 100084, China; wpq19@mails.tsinghua.edu.cn

† These authors contributed equally to this work.

* Correspondence: kunmingchen@nwsuaf.edu.cn or kunmingchen@163.com.cn; Tel.: +86-29-8703-1358; Fax: +86-29-8709-2262.

1. Supplementary Tables

Table S1. The detailed information about the members of NADPH oxidases (NOXs/RBOHs) gene family in plants

Name	Accession No.	AA.	Tissue specificity and function	Regulation	References
Arabidopsis thaliana					
AtRbohA	O81209[R]	902	Expression in root	Unknown	(Sagi and Fluhr, 2006)
AtRbohB	Q9SBI0[R]	843	Expression in germinating seeds.<br>Function: seed germination and endosperm weakening	ABA signaling; Post-translational protein modifications	(Muller et al., 2009)
AtRbohC	O81210[R]	905	Expression in root.<br>Function: root hair growth; uptake and translocation of Cd; antioxidant defenses; transport and translocation of As and macro/micronutrients	ROP GTPases; Ca^{2+}<br>Unknown<br>Unknown	(Foreman et al., 2003)<br>(Jones et al., 2007)<br>(Gupta et al., 2017)<br>(Gupta et al., 2013)
AtRbohD	Q9FIJ0[R]	921	Expression in whole plant.<br>Function: root development; immune response to fungus; involving in complex with EFR and FLS2; Initiation or prevention of cell death dependent on the distance from pathogen attack; trigger death in infection-damaged cells; inhibits death in neighboring cells; stimulate autophagosome formation and limited hypersensitive (HR) cell death; rapid closure of leaf stomata and enhance resistance to bacterial pathogen; rapid defense response activation improving salt tolerance by regulating Na^+/K^+ homeostasis and increase proline accumulation; mediate long-distance cell-to-cell communication; regulateing rapid systemic acquired acclimation (SAA) and systemic response to wound stimulis; abiotic stresses; hypoxia response; uptake and translocation of Cd	ABA;<br>Ca^{2+};<br>Auxin independent;<br>Pathogen- associated molecular patterns;<br>Salicylic acid;<br>Ethylene;<br>BIK1;<br>SA;<br>Purinergic signaling<br>CPK5;<br>Ethylene;<br>Hypoxia signaling;<br>H_2O_2 signaling	(Jiao et al., 2013)<br>(Li et al., 2015a)<br>(Morales et al., 2016)<br>(Foley et al., 2013)<br>(Pogany et al., 2009)<br>(Kadota et al., 2014)<br>(Liu et al., 2015)<br>(Chen et al., 2017)<br>(Dubiella et al., 2013)<br>(Ma et al., 2012)<br>(Deinlein et al., 2014)<br>(Ben Rejeb et al., 2015a)<br>(Ben Rejeb et al., 2015b)<br>(Miller et al., 2009)<br>(Mittler and Blumwald, 2015)<br>(Yang and Hong, 2015)<br>(Gupta et al., 2017)
AtRbohE	O81211[R]	952	Expression in stamens, tapetum.<br>Function: pollen development	Tapetal transcriptional network	(Xie et al., 2014)

Table S1 Continued

Name	Accession No.	AA.	Tissue specificity and function	Regulation	References
AtRbohF	O48538[R]	944	Expression in whole plant.<br>Function: root development; immune response to fungus; HR-related cell death; netabolomic responses and resistance; improving salt tolerance by regulating Na^+/K^+ homeostasis and increase proline accumulation; protect leaves from salinity stress by regulating xylem's loading of Na^+; uptake and translocation of Cd; cold responses.	Ca^{2+} ; Auxin independent;<br>Pathogen- associated molecular patterns;<br>Ethylene;<br>Na^+ accumulation; $[Ca^{2+}]$cyt elevation;<br>Binding to AtSRC2	(Foreman et al., 2003)<br>(Jones et al., 2007)<br>(Morales et al., 2016)<br>(Foley et al., 2013)<br>(Chaouch et al., 2012)<br>(Ma et al., 2012)<br>(Deinlein et al., 2014)<br>(Ben Rejeb et al., 2015a)<br>(Ben Rejeb et al., 2015b)<br>(Kurusu et al., 2015)<br>(Gupta et al., 2017)<br>(Kawarazaki et al., 2013)
AtRbohG	Q9SW17[R]	849	Expression in root	Unknown	(Sagi and Fluhr, 2006)
AtRbohH	Q9FJD6[R]	886	Expression in pollen tube.<br>Function: pollen tube tip growth.	Protein Phosphorylation; Ca^{2+}	(Kaya et al., 2014)<br>(Kaya et al., 2015)
AtRbohI	Q9SUT8[R]	941	Expression in root.<br>Function: hypoxic response, response to ethylene and hypoxia signaling.	Ethylene; Hypoxia signaling;<br>H_2O_2 signaling	(Lin et al., 2017)
AtRbohJ	Q9LZU9[R]	912	Expression in pollen tube.<br>Function: pollen tube tip growth.	Protein phosphorylation; Ca^{2+}	(Kaya et al., 2014)<br>(Kaya et al., 2015)
Brassica oleracea					
BoRbohD	D5LM24[U]	200	Expression in whole plant	Unknown	(Jakubowicz et al., 2010)
BoRbohF	D5LM19[U]	194	Expression in whole plant	Unknown	(Jakubowicz et al., 2010)
			Citrullus colocynthis		
CcRbohD	C1IHQ9[U]; EACF05505[G]	926	Expression in seeds. Function: drought tolerance; root development leaf morphogenesis.	Unknown	(Si et al., 2010)
Cucumis sativus					
CsRboh	B6DQ62[U]; A0A0A0LWE8[U]	889	Expression in seeds; root.<br>Function: brassinosteroid-induced stress tolerance; salt tolerance and regulating stomatal closure; response to cadmium stress.	MAPK cascade; H_2O_2 signaling pathway;<br>Brassinosteroid-induced pathway	(Xia et al., 2009)<br>(Niu et al., 2018)<br>(Jakubowska et al., 2015)<br>(Jakubowska and Janicka, 2017)

Table S1 Continued

Name	Accession No.	AA.	Tissue specificity and function	Regulation	References
Fragaria x ananassa					
FvRbohA	gene31855[#]	1027	Expression in root, stem, leaf, flower, and fruit. Function: cold stress tolerance.	Unknown	(Zhang et al., 2018)
FvRbohB	gene22214[#]	886	Expression in root, stem, leaf, flower, and fruit. Function: cold stress tolerance.	Unknown	(Zhang et al., 2018)
FvRbohC	gene01814[#]	1865	Expression in chloroplast thylakoid membrane, root, stem, leaf, flower, and fruit. Function: cold stress tolerance.	Unknown	(Zhang et al., 2018)
FvRbohD	gene00215[#]	935	Expression in root, stem, leaf, flower, and fruit. Function: cold stress tolerance.	Unknown	(Zhang et al., 2018)
FvRbohE	gene12928[#]	882	Expression in root, stem, leaf, flower, and fruit. Function: cold stress tolerance.	Unknown	(Zhang et al., 2018)
FvRbohF	gene26084[#]	791	Expression in root, stem, leaf, flower, and fruit. Function: cold stress tolerance.	Unknown	(Zhang et al., 2018)
FvRbohH	gene14024[#]	865	Expression in flower, and fruit. Function: cold stress tolerance.	Unknown	(Zhang et al., 2018)
***Glycine* max**					
GmNOX1	Gma01G222700[P]	927	Expressed in leaf.	Response to JA, SA, ABA treatment	(Zhang et al., 2019)
GmNOX2	Gma03G236300[P]	885	Expression in the root.	Up-regulated under PEG stress and JA, SA, ABA treatment	(Zhang et al., 2019)
GmNOX3	Gma04G203200[P]	928	Expressed in pod.	Unknown	(Zhang et al., 2019)
GmNOX4	Gma05G021100[P]	820	Expressed in root, early flower, stem and pod.	Unknown	(Zhang et al., 2019)
GmNOX5	Gma05G198700[P]	898	Expressed in flower, leaf, pod, and stem.	Up-regulated under PEG stress.	(Zhang et al., 2019)
GmNOX6	Gma05G212500[P]	941	Expression in stem, and pod.	Unknown	(Zhang et al., 2019)
GmNOX7	Gma06G162300[P]	941	Expressed in flower.	Unknown	(Zhang et al., 2019)
GmNOX8	Gma07G130800[P]	859	Expressed in flower.	Unknown	(Zhang et al., 2019)
GmNOX9	Gma08G005900[P]	888	Expressed in stem, and pod.	Unknown	(Zhang et al., 2019)
GmNOX10	Gma08G018900[P]	941	Expressed in root, seed, and flower.	Unknown	(Zhang et al., 2019)
GmNOX11	Gma09G073200[P]	928	Transcript accumulation in the early flower.	Unknown	(Zhang et al., 2019)
GmNOX12	Gma10G152200[P]	825	Expression in the root	Up-regulated under PEG stress and JA, SA, ABA treatment	(Zhang et al., 2019)

Table S1 Continued

Name	Accession No.	AA.	Tissue specificity and function	Regulation	References
GmNOX13	Gma11G020700[P]	927	Expression in leaf, flower, and pod.	Unknown	(Zhang et al., 2019)
GmNOX14	Gma15G182000[P]	936	Transcript accumulation in the early flower.	Unknown	(Zhang et al., 2019)
GmNOX15	Gma17G078300[P]	821	Expressed in root, early flower, stem and pod.	Unknown	(Zhang et al., 2019)
GmNOX16	Gma19G233900[P]	887	Expression in the root	Unknown	(Zhang et al., 2019)
GmNOX17	Gma20G236200[P]	889	Expression in the root	Up-regulated under PEG stress and JA, SA, ABA treatment	(Zhang et al., 2019)
Lepidium sativum					
LesaRbohA	JX312066[G]; J9WU02[U]	367	Expression in seed, root, leaf, cotyledons, bud, flower.	Unknown	(Müller et al., 2012)
LesaRbohB	JX312067[G]; J9WPU6[U]	810	Expression in seed, root, leaf, cotyledons, bud, flower. Function: root development, Auxin signaling.	Unknown	(Müller et al., 2012)
LesaRbohD	JX312068[G]; J9WLY1[U]	356	Expression in seed, root, leaf, cotyledons, bud, flower.	Unknown	(Müller et al., 2012)
LesaRbohF	JX312069[G]; J9WMN4[U]	943	Expression in seed, root, leaf, cotyledons, bud, flower.	Unknown	(Müller et al., 2012)
LesaRbohH	JX312070[G]		Expression in seed, root, leaf, cotyledons, bud, flower.	Unknown	(Müller et al., 2012)
Lycopersicon esculentum					
LeRboh1	Q9XEG2[U]	989	Expression in flowers, roots, eaves, fruits. Function: response to pathogen and wound.	Hormone	(Sagi et al., 2004)
LeWfi1	Q9LDD7[U]	938	Function: response to insect feeding and pathogen attack.	Hormone	(Sagi et al., 2004)
Malus × domestica					
MdRbohD1	MDP0000703059*	927	Expression in field grown trees, shoot and suspension culture cell. Function: shoot development and stress response.	Unknown	(Cepauskas et al., 2016)
MdRbohD2	MDP0000262620*	965	Expression in field grown trees, shoot and suspension culture cell. Function: shoot development and stress response.	Unknown	(Cepauskas et al., 2016)
MdRbohD3	MDP0000421679*	727	Expression in field grown trees, shoot and suspension culture cell.	Unknown	(Cepauskas et al., 2016)
MdRbohK	MDP0000280452*	816		Unknown	(Cepauskas et al., 2016)
MdRbohH1	MDP0000160005*	857	Expression in vitro shoots, leaves.	Unknown	(Cepauskas et al., 2016)

Table S1 Continued

Name	Accession No.	AA.	Tissue specificity and function	Regulation	References
MdRbohH2	MDP0000195681*	886	Expression in vitro shoots, suspension culture cell.	Unknown	(Cepauskas et al., 2016)
MdRbohE1	MDP0000264232*	1145		Unknown	(Cepauskas et al., 2016)
MdRbohE2	MDP0000273819*	1064	Expression in vitro shoots.	Unknown	(Cepauskas et al., 2016)
MdRbohF	MDP0000920069*	486	Expression in field grown trees. Function: shoot development and stress response.	Unknown	(Cepauskas et al., 2016)
Medicago truncatula					
MtRbohA	Medtr1g099800[P]	929	Expression in nodules, roots and root Hairs. Function: nodule nitrogen fixation.	Hypoxia	(Marino et al., 2011)
MtRbohB	Medtr3g151540[P]	923	Expression in stems, roots, root hairs, nodules, pods.	Unknown	(Marino et al., 2011)
MtRbohC	Medtr3g151570[P]	917	Expression in low expression. Function: root development, Auxin signaling.	Unknown	(Marino et al., 2011)
MtRbohD	Medtr3g151600[P]	729	Expression in roots, root hairs.	Unknown	(Marino et al., 2011)
MtRbohE	Medtr4g144710[P]	932	Expression in pods, flowers and stems.	Unknown	(Marino et al., 2011)
MtRbohF	Medtr7g067680[P]	849	Expression in root hairs and roots. Function: root hair development.	Unknown	(Marino et al., 2011)
MtRbohG	Medtr7g138940[P]	895	Expression in roots, stems, flowers, pods and leaves.	Unknown	(Marino et al., 2011)
Nicotiana attenuata					
NaRbohD	B8PS20[U]	937	Expression in leaves. Function: Herbivore-elicited responses.	JA	(Wu et al., 2013)
Nicotiana benthamiana					
NbRbohA	Q84KK8[U]	962	Expression in leaves. Function: participate in H_2O_2 Accumulation and pesistance to *Phytophthora infestans.*	Unknown	(Yoshioka et al., 2003)
NbRbohB	Q84KK7[U]	939	Expression in leaves. Function: Transient PTI ROS burst and robust ETI ROS burst.	MAPK pathway; Phosphatidic acid; CBL10-CIPK6 complex	(Yoshioka et al., 2003) (Yoshioka et al., 2016)
Nicotiana tabacum					
NtRbohD	Q8LRN5[U]	939	Expression in pollen tube. Function: pollen tube growth; plant defence reactions; response to fungals.	Ca^{2+} signaling; Phospholipids and Rac/Rop GTPases	(Potocky et al., 2007) (Potocky et al., 2012) (Lequeu et al., 2005) (Noirot et al., 2014)
NtRbohF	A7LP32[U]	962	Expression in pollen tube. Function: pollen tube growth.	Ca^{2+} signaling; Phospholipids and Rac/Rop GTPases	(Potocky et al., 2007) (Potocky et al., 2012)
NtNOX1	DQ497543[G]	200	Expression in nectar. Function: microoganisms defence.	Unknown	(Carter et al., 2007)

Table S1 Continued

Name	Accession No.	AA.	Tissue specificity and function	Regulation	References
Pyrus bretschneideri					
PbRBOHA	Pbr018609.1[GD]	962	Distributed on plasma membrane; Function: may play an important role in ROS metabolism during the lignification of pear stone cells.	Response to SA, ABA and MeJA.	(Cheng, et al., 2019)
PbRBOHB	Pbr037815.1[GD]	964	Function: may play crucial role during the lignification of pear stone cells	Response to SA, ABA and MeJA.	(Cheng, et al., 2019)
PbRBOHC	Pbr038667.1[GD]	947	Distributed on plasma membrane;	Response to SA, ABA and MeJA.	(Cheng, et al., 2019)
PbRBOHD	Pbr006277.1[GD]	978	Distributed in cytoplasm; Function: may play an important role in ROS metabolism during the lignification of pear stone cells.	Response to SA, ABA and MeJA.	(Cheng, et al., 2019)
PbRBOHE	Pbr036006.1[GD]	915	Distributed in cytoplasm;	Response to SA, ABA and MeJA.	(Cheng, et al., 2019)
PbRBOHF	Pbr003403.1[GD]	914	Distributed in cytoplasm;	Response to SA, ABA and MeJA.	(Cheng, et al., 2019)
PbRBOHG	Pbr023445.1[GD]	856	Distributed in cytoplasm;	Response to SA, ABA and MeJA.	(Cheng, et al., 2019)
PbRBOHH	Pbr033955.1[GD]	860	Distributed in cytoplasm;	Response to SA, ABA and MeJA.	(Cheng, et al., 2019)
PbRBOHI	Pbr007212.1[GD]	779	Distributed on plasma membrane;	Response to SA, ABA and MeJA.	(Cheng, et al., 2019)
PbRBOHJ	Pbr037399.1[GD]	701	Distributed in chloroplast;	Response to SA, ABA and MeJA.	(Cheng, et al., 2019)
Phaseolus vulgaris					
PvRbohA	Phvulv091020621m.g[P]	899	Expression in roots, leaves, shoots and nodules.<br>Function: enhancing *Rhizobium* infection and its release into the nodule cells.	Unknown	(Montiel et al., 2012)
PvRbohB	Phvulv091013731m.g[P]; H2E690[U]	881	Expression in shoots, roots, nodules; Low in leaves. Function: root growth and development, rhizobial invasion, nitrogen fixation and symbiotic process.	Unknown	(Montiel et al., 2012)<br>(Montiel et al., 2013)<br>(Arthikala et al., 2014)
PvRbohC	Phvulv091002467m.g[P]	930	Expression in roots, leaves, shoots and nodules.	Unknown	(Montiel et al., 2012)
PvRbohD	Phvulv091005473m.g[P]	890	Expression in shoots and leaves. Function: nodule nitrogen fixation.	Unknown	(Montiel et al., 2012)
PvRbohE	Phvulv091006195m.g[P]	926	Unknown	Unknown	(Montiel et al., 2012)
PvRbohF	Phvulv091020738m.g[P]	946	Function: root development, Auxin signaling.	Unknown	(Montiel et al., 2012)
PvRbohG	Phvulv091007712m.g[P]	823	Unknown	Unknown	(Montiel et al., 2012)

Table S1 Continued

Name	Accession No.	AA.	Tissue specificity and function	Regulation	References
PvRbohH	Phvulv091002934m.g[P]	914	Unknown	Unknown	(Montiel et al., 2012)
PvRbohI	Phvulv091023888m.g[P]	926	Expression in shoots. Function: root hair development.	Unknown	(Montiel et al., 2012)
Solanum tuberosum					
StRbohA	AB050660[G]	963	Expression in tubers.<br>Function: wound-induced oxidative burst/ microbial resistant, dormancy break.	GA/ABA	(Kumar et al., 2007)<br>(Liu et al., 2017)
StRbohB	AB050661[G]	867	Expression in leaves, sprouting tubers. Function: pathogen resistance and dormancy break	MAPK; GA/ABA	(Kumar et al., 2007)<br>(Yamamizo et al., 2007)<br>(Liu et al., 2017)<br>(Kobayashi et al., 2007)
StRbohC	AB198716[G]	938	Expression in leaves. Function: pathogen resistance.	MAPK cascade, MeJA	(Kumar et al., 2007)<br>(Yamamizo et al., 2007)
StRbohD	AB198717[G]	858	Expression in leaves. Function: pathogen resistance.	MAPK cascade, HWC, SA	(Kumar et al., 2007)<br>(Yamamizo et al., 2007)
StRbohF	AB064343[G]	944	Unknown	Unknown	(Kumar et al., 2007)
Vicia faba					
VfRbohA	G4XDS1[U]; JF784279[G]	894	Expression in cotyledons	Ethylene	(Andriunas et al., 2012)
VfRbohB	JF784281[G]	375	Unknown	Unknown	(Andriunas et al., 2012)
VfRbohC	G4XDS2[U];JF784280[G]	950	Unknown	Unknown	(Andriunas et al., 2012)
Vitis vinifera					
VvRbohA	XP_002277529.1[G];F6HUN7[U]	943	Expression in root; tendril; stem, leaf, ovule, inflorescence, berry.<br>Function: salt and drought response.	Unknown	(Cheng et al., 2013)
VvRbohB	XP_002283888.1[G];F6I4L7[U]	894	Expression in root; tendril; stem, leaf, ovule, inflorescence, berry; inflorescence.<br>Function: salt and drought response; powdery mildew inoculation.	Unknown	(Cheng et al., 2013)
VvRbohD	XP_002268641.1[G];	922	Expression in root; tendril; stem, leaf, ovule, inflorescence, berry; inflorescence.<br>Function: response to salicylic acid and powdery mildew.	Unknown	(Cheng et al., 2013)
VvRbohE	XP_002277540.1[G];	893	Expression in ovule, inflorescence, root, leaf, stem, tendril and berry.	Unknown	(Cheng et al., 2013)
VvRbohH	XP_002281695.1[G]	841	Expression in tendril, root, stem, inflorescence, leaf; berry and ovule.<br>Function: response to salicylic acid treatment.	Unknown	(Cheng et al., 2013)
Festuca arundinacea					
FRbohD	KF811502[G]	716	Expression in leave and root. Function: drought tolerance.	Unknown	(Rohollahi et al., 2018)

Table S1 Continued

Name	Accession No.	AA.	Tissue specificity and function	Regulation	References
Hordeum vulgare					
HvRbohA	A2VB85 [U]	963	Expression in leave. Function: response to pathogen; facilitation to fungal penetration.	Unknown	(Trujillo et al., 2006)
HvRbohB1	B2D0N5[U];EU566852[G]	845	Expression in immature head, root, coleoptile tissues.	Unknown	(Lightfoot et al., 2008)
HvRbohB2	B2D0N6[U];EU566853[G]	905	Expression in head, root and coleoptile; low in young leaf and flag leaf.	GA; ABA	(Lightfoot et al., 2008)<br>(Ishibashi et al., 2015)
HvRbohE	B2D0N7[U];EU566854[G]	986	Expression in head, root, coleoptile.	GA; ABA	(Lightfoot et al., 2008)
HvRbohB1	B2D0N5[U]; EU566852[G]	845	Expression in immature head, root, coleoptile tissues.	Unknown	(Lightfoot et al., 2008)
HvRbohB2	B2D0N6[U]; EU566853[G]	905	Expression in head, root and coleoptile; low in young leaf and flag leaf.	GA; ABA	(Lightfoot et al., 2008)<br>(Ishibashi et al., 2015)
HvRbohE	B2D0N7[U]; EU566854[G]	986	Expression in head, root, coleoptile.	GA; ABA	(Lightfoot et al., 2008)
HvRbohF1	B2D0N8[U]; EU566855[G]	946	Expression in root. Function: pathogen infection responses.	Unknown	(Lightfoot et al., 2008)
HvRbohF2	B2D0N9[U]; EU566856[G]	963	Expression in young and flag leaf. Function: pathogen infection responses.	GA; ABA	(Lightfoot et al., 2008)
HvRbohJ	B2D0P0[U]; EU566857[G]	843	Unknown	Unknown	(Lightfoot et al., 2008)
Olea europaea L.					
OeRbohH	KX648357G	833	Expression in olive pollen; Function: pollen germination and pollen tube growth.	High sensitivity to DPI (diphenylene iodonium chloride)	(Jimenez-Quesada et al., 2019)
Oryza sativa					
OsRbohA	Q0JJJ9[U]	743	Function: plant immune response; development regulation and drought tolerance; ABA-induced $\cdot O_2^-$ generation in the initial stage of leaf senescence.	Unknown	(Yoshie et al., 2005)<br>(Chang et al., 2016)<br>(Wang et al., 2016)<br>(Li et al., 2018)
OsRbohB	Q5ZAJ0[U]; BAS72095[G]	905	Function: plant immune response.	Ca^{2+}; Rac GTPase	(Yoshie et al., 2005)<br>(Wong et al., 2007)
OsRbohC	Q65XC8[U]; BAS95006[G]	951	Function: radicle and root elongation during seed germination; ABA-induced $\cdot O_2^-$ generation in the initial stage of leaf senescence.	Unknown	(Li et al., 2017)<br>(Li et al., 2018)
OsRbohD	Q0DHH6[U]; BAS94449[G]	819	Function: plant immune response.	Unknown	(Yoshie et al., 2005)

Table S1 Continued

Name	Accession No.	AA.	Tissue specificity and function	Regulation	References
OsRbohE	Q8S1T0[U]; BAS75100 [G]	843	Function: plant immune response and drought tolerance; regulate root hair initiation and elongation; ABA-induced $\cdot O_2^-$ generation in the initial stage of leaf senescence.	Unknown	(Yoshie et al., 2005) (Chang et al., 2016) (Li et al., 2018)
OsRbohF	Q0J595[U]; BAT05718[G]	1033	Function: defence response.	Unknown	(Yoshie et al., 2005)
OsRbohG	Q69LJ7[U]; BAT08263[G]	1007	Function: radicle and root elongation during seed germination; ABA-induced $\cdot O_2^-$ generation in the initial stage of leaf senescence.	Unknown	(Li et al., 2017) (Li et al., 2018)
OsRbohH	Q2QP56[U]; BAT17505[G]	909	Expression in roots; Function: ethylene-induced aerenchyma formation; radicle and root elongation during seed germination.	Interact with CDPK5/CDPK13	(Yamauchi et al., 2017) (Li et al., 2017)
OsRbohI	Q2R351[U];ABA94089[G]	936	Unknown	Unknown	(Chang et al., 2016)
Potamogeton crispus					
PcRboh1	Q9XEG3[U]	180	Unknown	Unknown	(Amicucci et al., 1999)
Triticum aestivum					
TaNOX1	TRIAE_CS42_1BL_TGACv1_030848_AA0102150.1[E]	832	Expression in function. Function:plant immune response.	Sensitive to biotic and abiotic stress; cold ↑	(Hu et al., 2018)
TaNOX2	TRIAE_CS42_1AL_TGACv1_002261_AA0040140.2[E]	972	Expression in whole plant. Function: plant growth regulation and stress responses.	Sensitive to biotic and abiotic stress and ABA, SA, MeJA treatment; NaCl ↑, cold ↑, drought ↑, heat ↑	(Hu et al., 2018)
TaNOX3	TRIAE_CS42_1DL_TGACv1_061195_AA0188450.1[E]	1160	Expression in root and elongation zone. Function: root development and seed after ripening.	Sensitive to biotic and abiotic stress and SA treatment; drought ↑, cold ↓	(Hu et al., 2018)
TaNOX5	TRIAE_CS42_3AL_TGACv1_195088_AA0644550.1[E]	851	Function: plant immune response.	Sensitive to biotic and abiotic stress and ABA treatment	(Hu et al., 2018)
TaNOX6	TGACv1_scaffold_195736_3AL:18346-27475[E]	976	Expression in stem.	Sensitive to biotic and abiotic stress; Development regulation; cold↑	(Hu et al., 2018)
TaNOX7	TGACv1_scaffold_211536_3AS:54051-73959[E]	901	Expression in seed. Function: response to biotic stresses and ABA treatment.	Sensitive to biotic and abiotic stress; ABA and MeJA treatment;	(Hu et al., 2018)
TaNOX8	TGACv1_scaffold_343050_4DL:40633-46817[E]	841	Expression in seed, stem. Function: response to biotic stresses and ABA treatment.	Sensitive to biotic and abiotic stress; heat↑	(Hu et al., 2018)
TaNOX9	TGACv1_scaffold_343029_4DL:55967-62975[E]	921	Unknown	Sensitive to biotic and abiotic stress; heat↑	(Hu et al., 2018)
TaNOX10	TGACv1_scaffold_404354_5BL:132580-133920[E]	918	Expression in whole plant (except seedling, seed, booting,).	Sensitive to biotic stress and MeJA treatment	(Hu et al., 2018)
TaNOX11	TGACv1_scaffold_375026_5AL:12006-19009[E]	919	Unknown	Sensitive to biotic stress	(Hu et al., 2018)

Table S1 Continued

Name	Accession No.	AA.	Tissue specificity and function	Regulation	References
TaNOX12	TGACv1_scaffold_406190_5BL:21326-32065[E]	985	Expression in stem.	Sensitive to biotic and abiotic stress, ABA treatment; drought↑, cold↑, NaCl↓	(Hu et al., 2018)
TaNOX13	TGACv1_scaffold_377290_5AL:27991-34187[E]	847	Expression in seed, stem. Function: response to biotic stresses and ABA treatment.	Sensitive to biotic and abiotic stress, ABA treatment; drought↑, cold↑	(Hu et al., 2018)
TaNOX14	TGACv1_scaffold_393979_5AS:10356-14798[E]	877	Expression in stem. Function: response to biotic stresses and ABA treatment.	Sensitive to biotic stress; Development regulation	(Hu et al., 2018)
TaNOX15	TGACv1_scaffold_513624_6BS:121044-129225[E]	957	Expression in seed. Function: root-hair-tip growth.	Unknown	(Hu et al., 2018)
TaNOX-like1	TGACv1_scaffold_001598_1AL:24546-36314[E]	651	Unknown	Unknown	(Hu et al., 2018)
TaNOX-like2	TGACv1_scaffold_030295_1BL:132356-145070[E]	675	Unknown	Unknown	(Hu et al., 2018)
TaNOX-like3	TGACv1_scaffold_032482_1BL:22766-37498[E]	602	Unknown	Unknown	(Hu et al., 2018)
TaNOX-like4	TGACv1_scaffold_542557_6DS:1-2175[E]	506	Unknown	Unknown	(Hu et al., 2018)
Zea mays					
ZmRbohA	Q0MRQ8[U]	852	Expression in leaves.	ABA, H_2O_2, MAPK	(Lin et al., 2009a)
ZmRbohB-α	DQ890023[G]	942	Expression in leaves, stems , roots, seedlings. Function: respond to NaCl, cold, heat, UV, wounding.	ABA, H_2O_2, MAPK	(Lin et al., 2009b)
ZmRbohB-β	C0J4N7 [U]; EU807966[G]	842	Expression in stems, leaves, roots, male flowers. Function: respond to NaCl, cold, heat, UV, wounding.	ABA, H_2O_2, MAPK	(Lin et al., 2009b)
ZmRbohC	A5H238[U]; DQ897930[G]	948	Unknown	ABA, H_2O_2, MAPK	(Lin et al., 2009a)
ZmRbohD	TGACv1_scaffold_195736_3AL:18346-27475[E]	648	Unknown	ABA, H_2O_2, MAPK	(Lin et al., 2009a)
ZmRbohE	GRMZM2G448185[P]; EU825209[G]	1009	Unknown	Unknown	(Montiel et al., 2012)
ZmRbohF	GRMZM2G300965[P]	931	Unknown	Unknown	(Montiel et al., 2012)
ZmRbohG	GRMZM2G037993[P]	882	Unknown	Unknown	(Montiel et al., 2012)
ZmRbohH	GRMZM2G323731[P]; AFW56509[G]	822	Unknown	Unknown	(Montiel et al., 2012)
ZmRbohI	GRMZM2G022547[P]	897	Unknown	Unknown	(Montiel et al., 2012)
ZmRbohJ	GRMZM2G065144[P]	948	Unknown	Unknown	(Montiel et al., 2012)
ZmRbohK	GRMZM2G358619[P]	976	Unknown	Unknown	(Montiel et al., 2012)

Table S1 Continued

Name	Accession No.	AA.	Tissue specificity and function	Regulation	References
RTH5	GRMZM2G426953[G]	Unknown	Expression in epidermal cells of the primary root. Function: root hair initiation and elongation.	Unknown	(Nestler et al., 2014)
Chondrus crispus					
CcRboh	Q2FA46[U]	825	Function: response to pathogen/MeJA/Atrazine/Metolachlor/H_2O_2	Unknown	(Herve´ et al., 2006)
Cyanidioschyzon merolae					
CmRboh1	M1VG56[U]	864	Unknown	Unknown	(Herve´ et al., 2006)
CmRboh2	M1UWF8[U]	935	Unknown	Unknown	(Herve´ et al., 2006)
Porphyria yezoensis					
PyRboh	Q2F9N3[U]	903	Unknown	Unknown	(Herve´ et al., 2006)

AA.: amino acid; [R] Reviewed UniProt IDs; [U] Unreviewed UniProt IDs; [E] Ensembl Plants IDs; [P] Phytozome IDs; [G] GenBank; *Sequence From Apple Genome; #Genome Database for Rosaceae; [GD] GIGADB Databases ↑ Up Regulated; ↓ Down Regulated

Reference

Amicucci, E., Gaschler, K., and Ward, J.M. (1999). NADPH oxidase genes from tomato (*Lycopersicon esculentum*) and curly-leaf pondweed (*Potamogeton crispus*). Plant Biology 1:524-528.

Andriunas, F., Zhang, H., Xia, X., Offler, C., McCurdy, D., and Patrick, J. (2012). Reactive oxygen species form part of a regulatory pathway initiating trans-differentiation of epidermal transfer cells in *Vicia faba* cotyledons. J. Exp. Bot. 63:3617-3629.

Arthikala, M.K., Sanchez-Lopez, R., Nava, N., Santana, O., Cardenas, L., and Quinto, C. (2014). *RbohB*, a *Phaseolus vulgaris* NADPH oxidase gene, enhances symbiosome number, bacteroid size, and nitrogen fixation in nodules and impairs mycorrhizal colonization. New Phytol. 202:886-900.

Ben Rejeb, K., Benzarti, M., Debez, A., Bailly, C., Savoure, A., and Abdelly, C. (2015a). NADPH oxidase-dependent H_2O_2 production is required for salt-induced antioxidant defense in *Arabidopsis thaliana*. J. Plant Physiol. 174:5-15.

Ben Rejeb, K., Lefebvre-De Vos, D., Le Disquet, I., Leprince, A.S., Bordenave, M., Maldiney, R., Jdey, A., Abdelly, C., and Savoure, A. (2015b). Hydrogen peroxide produced by NADPH oxidases increases proline accumulation during salt or mannitol stress in *Arabidopsis thaliana*. New Phytol. 208:1138-1148.

Carter, C., Healy, R., O'Tool, N.M., Naqvi, S.M., Ren, G., Park, S., Beattie, G.A., Horner, H.T., and Thornburg, R.W. (2007). Tobacco nectaries express a novel NADPH oxidase implicated in the defense of floral reproductive tissues against microorganisms. Plant Physiol. 143:389-399.

Cepauskas, D., Miliute, I., Staniene, G., Gelvonauskiene, D., Stanys, V., Jesaitis, A.J., and Baniulis, D. (2016). Characterization of apple NADPH oxidase genes and their expression associated with oxidative stress in shoot culture in vitro. Plant Cell Tiss. Org. 124:621-633.

Chang, Y.L., Li, W.Y., Miao, H., Yang, S.Q., Li, R., Wang, X., Li, W.Q., and Chen, K.M. (2016). Comprehensive Genomic Analysis and Expression Profiling of the NOX Gene Families under Abiotic Stresses and Hormones in Plants. Genome Biol. Evol 8:791-810.

Chaouch, S., Queval, G., and Noctor, G. (2012). AtRbohF is a crucial modulator of defence-associated metabolism and a key actor in the interplay between intracellular oxidative stress and pathogenesis responses in Arabidopsis. Plant J. 69:613-627.

Chen, D., Cao, Y., Li, H., Kim, D., Ahsan, N., Thelen, J., and Stacey, G. (2017). Extracellular ATP elicits DORN1-mediated RBOHD phosphorylation to regulate stomatal aperture. Nat. Commun. 8:2265.

Cheng, C., Xu, X., Gao, M., Li, J., Guo, C., Song, J., and Wang, X. (2013). Genome-wide analysis of respiratory burst oxidase homologs in grape (*Vitis vinifera* L.). Int. J. Mol. Sci. 14:24169-24186.

Cheng, X., Li, G., Manzoor, M. A., Wang, H., Abdullah, M., Su, X., Zhang, J., Jiang, T., Jin, Q., Cai, Y., and Lin, Y. (2019). In Silico Genome-Wide Analysis of Respiratory Burst Oxidase Homolog (Rboh) Family Genes in Five Fruit-Producing Trees, and Potential Functional Analysis on Lignification of Stone Cells in Chinese White Pear. Cells 8: 520.

Deinlein, U., Stephan, A.B., Horie, T., Luo, W., Xu, G., and Schroeder, J.I. (2014). Plant salt-tolerance mechanisms. Trends Plant Sci. 19:371-379.

Dubiella, U., Seybold, H., Durian, G., Komander, E., Lassig, R., Witte, C.P., Schulze, W.X., and Romeis, T. (2013). Calcium-dependent protein kinase/NADPH oxidase activation circuit is required for rapid defense signal propagation. Proc. Natl Acad. Sci. USA 110:8744-8749.

Foley, R.C., Gleason, C.A., Anderson, J.P., Hamann, T., and Singh, K.B. (2013). Genetic and genomic analysis of *Rhizoctonia solani* interactions with Arabidopsis; evidence of resistance mediated through NADPH oxidases. PloS one 8:e56814.

Foreman, J., Demidchik, V., Bothwell, J.H., Mylona, P., Miedema, H., Torres, M.A., Linstead, P., Costa, S., Brownlee, C., Jones, J.D., et al. (2003). Reactive oxygen species produced by NADPH oxidase regulate plant cell growth. Nature 422:442-446.

Gupta, D.K., Inouhe, M., Rodriguez-Serrano, M., Romero-Puertas, M.C., and Sandalio, L.M. (2013). Oxidative stress and arsenic toxicity: role of NADPH oxidases. Chemosphere 90:1987-1996.

Gupta, D.K., Pena, L.B., Romero-Puertas, M.C., Hernandez, A., Inouhe, M., and Sandalio, L.M. (2017). NADPH oxidases differentially regulate ROS metabolism and nutrient uptake under cadmium toxicity. Plant Cell Environ. 40:509-526.

Herve´, C., Tonon, T., Colle´n, J., Corre, E., and Boyen, C. (2006). NADPH oxidases in Eukaryotes: red algae provide new hints! Curr. Genet. 49:190-204.

Hu, C., Wei, X., Yuan, B., Yao, L., Ma, T., Zhang, P., Wang, X., Wang, P., Liu, W., Li, W., et al. (2018). Genome-wide identification and functional analysis of NADPH oxidase family genes in wheat during development and environmental stress responses. Front. Plant Sci. 9:906.

Ishibashi, Y., Kasa, S., Sakamoto, M., Aoki, N., Kai, K., Yuasa, T., Hanada, A., Yamaguchi, S., and Iwaya-Inoue, M. (2015). A role for reactive oxygen species produced by NADPH oxidases in the embryo and aleurone cells in barley seed germination. PloS one 10:e0143173.

Jakubowicz, M., Galganska, H., Nowak, W., and Sadowski, J. (2010). Exogenously induced expression of ethylene biosynthesis, ethylene perception, phospholipase D, and Rboh-oxidase genes in broccoli seedlings. J. Exp. Bot. 61:3475-3491.

Jakubowska, D., Janicka-Russak, M., Kabala, K., Migocka, M., and Reda, M. (2015). Modification of plasma membrane NADPH oxidase activity in cucumber seedling roots in response to cadmium stress. Plant Sci. 234:50-59.

Jakubowska, D., and Janicka, M. (2017). The role of brassinosteroids in the regulation of the plasma membrane H^+-ATPase and NADPH oxidase under cadmium stress. Plant Sci. 264:37-47.

Jiao, Y., Sun, L., Song, Y., Wang, L., Liu, L., Zhang, L., Liu, B., Li, N., Miao, C., and Hao, F. (2013). AtrbohD and AtrbohF positively regulate abscisic acid-inhibited primary root growth by affecting Ca^{2+} signalling and auxin response of roots in *Arabidopsis*. J. Exp. Bot. 64:4183-4192.

Jimenez-Quesada, M. J., Traverso, J. A., Potocky, M., Zarsky,V. and Alche J. D.(2019). Generation of Superoxide by Oerbohh, a Nadph Oxidase Activity During Olive (Olea Europaea L.) Pollen Development and Germination." Front Plant Sci 10: 1149.

Jones, M.A., Raymond, M.J., Yang, Z., and Smirnoff, N. (2007). NADPH oxidase-dependent reactive oxygen species formation required for root hair growth depends on ROP GTPase. J. Exp. Bot. 58:1261-1270.

Kadota, Y., Sklenar, J., Derbyshire, P., Stransfeld, L., Asai, S., Ntoukakis, V., Jones, J.D., Shirasu,

K., Menke, F., Jones, A., et al. (2014). Direct regulation of the NADPH oxidase RBOHD by the PRR-associated kinase BIK1 during plant immunity. Mol. Cell 54:43-55.

Kawarazaki, T., Kimura, S., Iizuka, A., Hanamata, S., Nibori, H., Michikawa, M., Imai, A., Abe, M., Kaya, H., and Kuchitsu, K. (2013). A low temperature-inducible protein AtSRC2 enhances the ROS-producing activity of NADPH oxidase AtRbohF. Biochim. Biophys. Acta. 1833:2775-2780.

Kaya, H., Iwano, M., Takeda, S., Kanaoka, M.M., Kimura, S., Abe, M., and Kuchitsu, K. (2015). Apoplastic ROS production upon pollination by RbohH and RbohJ in Arabidopsis. Plant Signal. Behav. 10:e989050.

Kaya, H., Nakajima, R., Iwano, M., Kanaoka, M.M., Kimura, S., Takeda, S., Kawarazaki, T., Senzaki, E., Hamamura, Y., Higashiyama, T., et al. (2014). Ca^{2+}-activated reactive oxygen species production by Arabidopsis RbohH and RbohJ is essential for proper pollen tube tip growth. The Plant cell 26:1069-1080.

Kobayashi, M., Ohura, I., Kawakita, K., Yokota, N., Fujiwara, M., Shimamoto, K., Doke, N., and Yoshioka, H. (2007). Calcium-dependent protein kinases regulate the production of reactive oxygen species by potato NADPH oxidase. The Plant cell 19:1065-1080.

Kumar, G.N., Iyer, S., and Knowles, N.R. (2007). Strboh A homologue of NADPH oxidase regulates wound-induced oxidative burst and facilitates wound-healing in potato tubers. Planta 227:25-36.

Kurusu, T., Kuchitsu, K., and Tada, Y. (2015). Plant signaling networks involving Ca^{2+} and Rboh/Nox-mediated ROS production under salinity stress. Front. Plant Sci. 6:8.

Lequeu, J., Simon-Plas, F., Fromentin, J., Etienne, P., Petitot, A.S., Blein, J.P., and Suty, L. (2005). Proteasome comprising a beta1 inducible subunit acts as a negative regulator of NADPH oxidase during elicitation of plant defense reactions. FEBS Lett 579:4879-4886.

Li, N., Sun, L., Zhang, L., Song, Y., Hu, P., Li, C., and Hao, F.S. (2015a). AtrbohD and AtrbohF negatively regulate lateral root development by changing the localized accumulation of superoxide in primary roots of Arabidopsis. Planta 241:591-602.

Li, W.Y., Chen, B.X., Chen, Z.J., Gao, Y.T., Chen, Z., and Liu, J. (2017). Reactive oxygen species generated by NADPH oxidases promote radicle protrusion and root elongation during rice seed germination. Int. J. Mol. Sci. 18:E110.

Li, Z., Wang, F., Zhao, Q., Liu, J., and Cheng, F. (2018). Involvement of NADPH oxidase isoforms in the production of O_2- manipulated by ABA in the senescing leaves of early-senescence-leaf (*esl*) mutant rice (*Oryza sativa*). PloS one 13:e0190161.

Lightfoot, D., Boettcher, A., Little, A., Shirley, N., and Able, A. (2008). Identification and characterisation of barley (*Hordeum vulgare*) respiratory burst oxidase homologue family members. Funct. Plant Biol. 35:347-359.

Lin, F., Ding, H., Wang, J., Zhang, H., Zhang, A., Zhang, Y., Tan, M., Dong, W., and Jiang, M. (2009a). Positive feedback regulation of maize NADPH oxidase by mitogen-activated protein kinase cascade in abscisic acid signalling. J. Exp. Bot. 60:3221-3238.

Lin, F., Zhang, Y., and Jiang, M.Y. (2009b). Alternative splicing and differential expression of two transcripts of nicotine adenine dinucleotide phosphate oxidase B gene from *Zea mays*. J. Integr. Plant Biol. 51:287-298.

Lin, I.S., Wu, Y.-S., Chen, C.-T., Chen, G.-H., Hwang, S.-G., Jauh, G.-Y., Tzen, J.T.C., and Yang, C.-Y. (2017). AtRBOH I confers submergence tolerance and is involved in auxin-mediated

signaling pathways under hypoxic stress. Plant Growth Regul. 83:277-285.

Liu, B., Zhao, S., Tan, F., Zhao, H., Wang, D., Si, H., and Chen, Q. (2017). Changes in ROS production and antioxidant capacity during tuber sprouting in potato. Food Chem. 237:205-213.

Liu, H.B., Wang, X.D., Zhang, Y.Y., Dong, J.J., Ma, C., and Chen, W.L. (2015). NADPH oxidase RBOHD contributes to autophagy and hypersensitive cell death during the plant defense response in *Arabidopsis thaliana*. Biol. Plantarum 59:570-580.

Ma, L., Zhang, H., Sun, L., Jiao, Y., Zhang, G., Miao, C., and Hao, F. (2012). NADPH oxidase AtrbohD and AtrbohF function in ROS-dependent regulation of Na^+/K^+homeostasis in *Arabidopsis* under salt stress. J. Exp. Bot. 63:305-317.

Marino, D., Andrio, E., Danchin, E.G.J., Oger, E., Gucciardo, S., Lambert, A., Puppo, A., and Pauly, N. (2011). A *Medicago truncatula* NADPH oxidase is involved in symbiotic nodule functioning. New Phytol. 189:580-592.

Miller, G., Schlauch, K., Tam, R., Cortes, D., Torres, M.A., Shulaev, V., Dangl, J.L., and Mittler, R. (2009). The plant NADPH oxidase RBOHD mediates rapid systemic signaling in response to diverse stimuli. Sci. Signal 2:ra45.

Mittler, R., and Blumwald, E. (2015). The roles of ROS and ABA in systemic acquired acclimation. The Plant cell 27:64-70.

Montiel, J., Arthikala, M.K., and Quinto, C. (2013). *Phaseolus vulgaris* RbohB functions in lateral root development. Plant Signal. Behav. 8:e22694.

Montiel, J., Nava, N., Cardenas, L., Sanchez-Lopez, R., Arthikala, M.K., Santana, O., Sanchez, F., and Quinto, C. (2012). A *Phaseolus vulgaris* NADPH oxidase gene is required for root infection by Rhizobia. Plant Cell Physiol. 53:1751-1767.

Morales, J., Kadota, Y., Zipfel, C., Molina, A., and Torres, M.A. (2016). The Arabidopsis NADPH oxidases *RbohD* and *RbohF* display differential expression patterns and contributions during plant immunity. J. Exp. Bot. 67:1663-1676.

Müller, K., Carstens, A.C., Linkies, A., Torres, M.A., and Leubner-Metzger, G. (2009). The NADPH-oxidase *AtrbohB* plays a role in Arabidopsis seed after-ripening. New Phytol. 184:885-897.

Müller, K., Linkies, A., Leubner-Metzger, G., and Kermode, A.R. (2012). Role of a respiratory burst oxidase of *Lepidium sativum* (cress) seedlings in root development and auxin signalling. J. Exp. Bot. 63:6325-6334.

Nestler, J., Liu, S.Z., Wen, T.J., Paschold, A., Marcon, C., Tang, H.M., Li, D.L., Li, L., Meeley, R.B., Sakai, H., et al. (2014). *Roothairless5*, which functions in maize (*Zea mays* L.) root hair initiation and elongation encodes a monocot-specific NADPH oxidase. Plant J. 79:729-740.

Niu, M., Huang, Y., Sun, S., Sun, J., Cao, H., Shabala, S., and Bie, Z. (2018). Root respiratory burst oxidase homologue-dependent H_2O_2 production confers salt tolerance on a grafted cucumber by controlling Na^+ exclusion and stomatal closure. J. Exp. Bot. 69:3465-3476.

Noirot, E., Der, C., Lherminier, J., Robert, F., Moricova, P., Kieu, K., Leborgne-Castel, N., Simon-Plas, F., and Bouhidel, K. (2014). Dynamic changes in the subcellular distribution of the tobacco ROS-producing enzyme RBOHD in response to the oomycete elicitor cryptogein. J. Exp. Bot. 65:5011-5022.

Nordzieke, D. E., T. R. Fernandes, M. El Ghalid, D. Turra, and A. Di Pietro (2019). Nadph Oxidase Regulates Chemotropic Growth of the Fungal Pathogen Fusarium Oxysporum Towards the

Host Plant. New Phytol 224, no. 4: 1600-12.

Pogany, M., von Rad, U., Grun, S., Dongo, A., Pintye, A., Simoneau, P., Bahnweg, G., Kiss, L., Barna, B., and Durner, J. (2009). Dual roles of reactive oxygen species and NADPH oxidase RBOHD in an Arabidopsis-*Alternaria* pathosystem. Plant Physiol. 151:1459-1475.

Potocky, M., Jones, M.A., Bezvoda, R., Smirnoff, N., and Zarsky, V. (2007). Reactive oxygen species produced by NADPH oxidase are involved in pollen tube growth. New Phytol. 174:742-751.

Potocky, M., Pejchar, P., Gutkowska, M., Jimenez-Quesada, M.J., Potocka, A., Alche Jde, D., Kost, B., and Zarsky, V. (2012). NADPH oxidase activity in pollen tubes is affected by calcium ions, signaling phospholipids and Rac/Rop GTPases. J. Plant Physiol. 169:1654-1663.

Rohollahi, I., Khoshkholghsima, N., Nagano, H., Hoshino, Y., and Yamada, T. (2018). *Respiratory burst oxidase-D* expression and biochemical responses in *Festuca arundinacea* under drought stress. Crop Sci. 58.

Sagi, M., Davydov, O., Orazova, S., Yesbergenova, Z., Ophir, R., Stratmann, J.W., and Fluhr, R. (2004). Plant respiratory burst oxidase homologs impinge on wound responsiveness and development in *Lycopersicon esculentum*. The Plant cell 16:616-628.

Sagi, M., and Fluhr, R. (2006). Production of reactive oxygen species by plant NADPH oxidases. Plant Physiol. 141:336-340.

Si, Y., Dane, F., Rashotte, A., Kang, K., and Singh, N.K. (2010). Cloning and expression analysis of the *Ccrboh* gene encoding respiratory burst oxidase in *Citrullus colocynthis* and grafting onto *Citrullus lanatus* (watermelon). J. Exp. Bot. 61:1635-1642.

Trujillo, M., Altschmied, L., Schweizer, P., Kogel, K.H., and Huckelhoven, R. (2006). *Respiratory burst oxidase homologue A* of barley contributes to penetration by the powdery mildew fungus *Blumeria graminis* f. sp. *hordei*. J. Exp. Bot. 57:3781-3791.

Wang, X., Zhang, M.M., Wang, Y.J., Gao, Y.T., Li, R., Wang, G.F., Li, W.Q., Liu, W.T., and Chen, K.M. (2016). The plasma membrane NADPH oxidase OsRbohA plays a crucial role in developmental regulation and drought-stress response in rice. Physiol. Plant. 156:421-443.

Wong, H.L., Pinontoan, R., Hayashi, K., Tabata, R., Yaeno, T., Hasegawa, K., Kojima, C., Yoshioka, H., Iba, K., Kawasaki, T., et al. (2007). Regulation of rice NADPH oxidase by binding of Rac GTPase to its N-terminal extension. The Plant cell 19:4022-4034.

Wu, J., Wang, L., Wunsche, H., and Baldwin, I.T. (2013). *Narboh D*, a respiratory burst oxidase homolog in *Nicotiana attenuata*, is required for late defense responses after herbivore attack. J. Integr. Plant Biol. 55:187-198.

Xia, X.J., Wang, Y.J., Zhou, Y.H., Tao, Y., Mao, W.H., Shi, K., Asami, T., Chen, Z., and Yu, J.Q. (2009). Reactive oxygen species are involved in brassinosteroid-induced stress tolerance in cucumber. Plant Physiol. 150:801-814.

Xie, H.T., Wan, Z.Y., Li, S., and Zhang, Y. (2014). Spatiotemporal production of reactive oxygen species by NADPH oxidase is critical for tapetal programmed cell death and pollen development in *Arabidopsis*. The Plant cell 26:2007-2023.

Yamamizo, C., Doke, N., Yoshioka, H., and Kawakita, K. (2007). Involvement of mitogen-activated protein kinase in the induction of *StrbohC* and *StrbohD* genes in response to pathogen signals in potato. J. Gen. Plant Pathol. 73:304-313.

Yamauchi, T., Yoshioka, M., Fukazawa, A., Mori, H., Nishizawa, N.K., Tsutsumi, N., Yoshioka, H., and Nakazono, M. (2017). An NADPH oxidase RBOH functions in rice roots during

Lysigenous Aerenchyma formation under oxygen-deficient conditions. The Plant cell 29:775-790.

Yang, C.Y., and Hong, C.P. (2015). The NADPH oxidase Rboh D is involved in primary hypoxia signalling and modulates expression of hypoxia-inducible genes under hypoxic stress. Environ. Exp. Bot. 115:63-72.

Yoshie, Y., Goto, K., Takai, R., Lwano, M., Takayama, S., Lsogai, A., and Che, F. (2005). Function of the rice gp91^{phox} homologs *OsrbohA* and *OsrbohE* genes in ROS-dependent plant immune responses. Plant Biotechnol. 22:127-135.

Yoshioka, H., Adachi, H., Nakano, T., Miyagawa, N., Asai, S., Ishihama, N., and Yoshioka, M. (2016). Hierarchical regulation of NADPH oxidase by protein kinases in plant immunity. Physiol. Mol. Plant P. 95:20-26.

Yoshioka, H., Numata, N., Nakajima, K., Katou, S., Kawakita, K., Rowland, O., Jones, J.D.G., and Doke, N. (2003). *Nicotiana benthamiana* gp91^{phox} homologs *NbrbohA* and *NbrbohB* participate in H_2O_2 accumulation and resistance to *Phytophthora infestans*. The Plant cell 15:706-718.

Zhang, Y., Li, Y., He, Y., Hu, W., Zhang, Y., Wang, X., and Tang, H. (2018). Identification of NADPH oxidase family members associated with cold stress in strawberry. FEBS Open Bio. 8:593-605.

Zhang, Z. B., Zhao,Y. L., Feng, X.B., Luo, Z. Y., Kong, S. W., Zhang, C., Gong, A. D., Yuan, H. Y., Cheng, L., and Wang, X. N. (2019). Genomic, molecular evolution, and expression analysis of NOX genes in soybean (*Glycine* max). Genomics, 111: 619-628.
